# Determination of Pyrethroids in Tea Brew by GC-MS Combined with SPME with Multiwalled Carbon Nanotube Coated Fiber

**DOI:** 10.1155/2018/8426598

**Published:** 2018-03-08

**Authors:** Dongxia Ren, Chengjun Sun, Guanqun Ma, Danni Yang, Chen Zhou, Jiayu Xie, Yongxin Li

**Affiliations:** ^1^West China School of Public Health, Sichuan University, Chengdu 610041, China; ^2^Provincial Key Laboratory for Food Safety Monitoring and Risk Assessment of Sichuan, Chengdu 610041, China; ^3^College of Life and Environmental Sciences, Shanghai Normal University, Shanghai, China

## Abstract

A new method has been developed to simultaneously determine 7 pyrethroid residues in tea brew using gas chromatography-mass spectrometry (GC-MS) combined with solid phase microextraction (SPME) with multiwalled carbon nanotubes (MWCNTs) coated fiber. The MWCNTs coated fiber of SPME was homemade by using stainless steel wire as coating carrier and polyacrylonitrile (PAN) solution as adhesive glue. Under the optimized conditions, a good linearity was shown for bifenthrin, fenpropathrin, permethrin, and cyfluthrin in 1–50 ng mL^−1^ and for cypermethrin, fenvalerate, and deltamethrin in 5–50 ng mL^−1^. The correlation coefficients were in the range of 0.9948–0.9999. The average recoveries of 7 pyrethroids were 94.2%–107.3% and the relative standard deviations (RSDs) were less than 15%. The detection limit of the method ranged from 0.12 to 1.65 ng mL^−1^. The tea brew samples made from some commercial tea samples were analyzed. Among them, bifenthrin, fenpropathrin, and permethrin were found. The results show that the method is rapid and sensitive and requires low organic reagent consumption, which can be well used for the detection of the pyrethroids in tea brew.

## 1. Introduction

Synthetic pyrethroids are a class of bionic synthetic ester pesticides whose structural or biological activity is similar to natural pyrethroids, with the features of high efficiency, broad spectrum, and less residue. Despite their low toxicity, long-term exposure and ingestion of these pesticides may result in endocrine disorders and affect nervous system function [1]. China is the homeland of tea, and tea has several beneficial functions, such as refreshing and relieving restlessness. Nowadays, people not only require better quality of tea, but also pay particular attention to the harmful substances in it [2]. Since tea is susceptible to suffer from pests and diseases, farmers usually spray pesticides to reduce the adverse effects, which results in pesticide residues [3–5]. The level of pesticide residues in tea brew is directly related to the intake amount of human, while the determination of these 7 pyrethroids in tea brew has rarely been reported [6, 7]. Therefore, it is necessary to develop a rapid and efficient method for the detection of these 7 pyrethroid pesticides in tea brew.

Recently, it is in tea that most researches are mainly focused on the detection of pyrethroid residues [2, 8]. Such methods as solid phase extraction, gel permeation chromatography, and matrix solid phase dispersion are commonly used as sample preparation techniques for analysis of pesticide residues in tea [9, 10]. However, organic solvents are employed during the extraction procedure, which poses a threat to the environment and human health. SPME is a new method of microextraction without solvent based on solid phase extraction, which has the advantages of simultaneous sampling, extraction and concentration, and low consumption of sample and reagents, as well as ease of operation and automation [8, 11]. Extraction phase is the core of SPME, whose performance directly determines the SPME efficiency, thus affecting the sensitivity of the method and the reliability of the results. However, the commercial fibers tend to swell off, and most of them use fused silica as the fiber carrier, which is expensive, is easy to break, and has short service life. In addition, the assortment of commercially available extraction phases is quite limited, which also limits their application. In order to improve the SPME performance, many researchers are committed to the research on new fibers.

In recent years, the emergence of many kinds of materials has brought new opportunities for the development of SPME fiber coating. Wang et al. used dispersive solid phase extraction with polyaniline-coated magnetic particles to determine 5 pyrethroids in tea drinks [12]. Sun et al. determined the pollutants in the aquatic environment by SPME coupled with surface enhanced Raman spectroscopy, in which the SPME was coated with ZnO nanorods (ZnO NRs) decorated with Au@4-ATP@Ag core–shell nanoparticles (NPs) [13]. Wang et al. synthesized a core−shell TiO_2_@C fiber for SPME to detect polycyclic aromatic hydrocarbons in river water samples [14]. Wu et al. prepared a single-walled carbon nanotubes coated SPME fiber for extraction of several pyrethroids [15]. Among these nanomaterials, MWCNTs, a new kind of carbon nanomaterials, have attracted much attention in many fields. As new adsorbents, MWCNTs present a promising future in the preconcentration of environmental contaminants, due to their unique hydrophobic structure and large specific surface area, as well as strong reaction with a variety of organic compounds [16, 17].

Up to now, the detection of these 7 pyrethroids in tea brew is rarely reported [6, 7]. In this study, a SPME with MWCNTs coated fiber was developed using stainless steel wire as the coating carrier, which in combination with GC-MS was applied to the detection of the target pyrethroids in tea brew with satisfactory results.

## 2. Experimental

### 2.1. Chemicals and Materials

The standards of bifenthrin, fenpropathrin, permethrin, cyfluthrin, cypermethrin, fenvalerate, and deltamethrin were purchased from National Standard Material Center of China. n-Hexane, methanol, and acetone were of HPLC grade and provided by Tianjin Branch Miou Chemical Reagent Co., Ltd. MWCNTs (10–30 *μ*m length, 10–20 nm outer diameter) were purchased from Chinese Academy of Sciences Chengdu Organic Chemistry Co., Ltd. N,N-Dimethylformamide (DMF) was obtained from Shanghai Guoyao Chemical Reagent Co., Ltd. Polyacrylonitrile (PAN) was purchased from Sigma (St. Louis, MO, USA). Water used throughout the experiments was obtained from a Millipore water purification system (18.25 M Ω cm, Millipore, USA).

Stainless steel wires (12 cm × 0.15 mm) were obtained from Shenzhen Santk Metal Material Co., Ltd. SPME needles (SPME-S-02) were purchased from Shanghai New Extension Analytical Instruments Technology Co., Ltd. SPME handle (57330-U) was obtained from Supelco.

### 2.2. Preparation of the Standard Solutions

The stock solutions of 100 *μ*g mL^−1^ were prepared with pyrethroid standards containing bifenthrin, fenpropathrin, permethrin, cyfluthrin, cypermethrin, fenvalerate, and deltamethrin in n-hexane and stored at −20°C. Five levels of calibration scales were obtained by diluting the stock standard solutions with n-hexane, which were stored at 4°C until used.

### 2.3. Preparation of MWCTs Coated Fiber for SPME

The stainless steel wires were cleaned with methanol, acetone, and n-hexane in order to remove the surface contaminants and then dried in the air at room temperature. Meanwhile, 0.5 mg of PAN was dissolved in 7.5 mL of DMF, and the mixture was kept at 90°C for 1 h. Five mg of MWCNTs was taken into a centrifuge tube and mixed with PAN solution by ultrasound. Subsequently, the cleaned stainless steel wire was dipped into the coating suspension with a height of 1.5 cm for one minute [18–20] and then withdrew at a constant rate to ensure the formation of a smooth coating on the wire surface. After this, the fiber was dried at 180°C for 2 min. This above procedure from “dipped into the coating suspension” was repeated 3 times. Prior to use, the proposed SPME was conditioned at 280°C for 2 h.

### 2.4. Sample Preparation

The tea samples were randomly purchased from the local markets. After fully mixed, 3 g (accurate to 0.001 g) of each was weighed into a 250 mL beaker and 100 mL of boiled water was added and then kept for 3 h. 10 mL of the above-mentioned supernatant in a vial with cap was placed on a magnetic stirrer and extracted with the proposed SPME with MWCNTs coated fiber at 750 rpm for 20 min. The target compounds were desorbed at 280°C for 8 min and determined by GC-MS.

### 2.5. GC-MS Analysis

GC-MS analysis was performed by Agilent Technologies 7890A GC with a 5975C MS, equipped with a DB-5 MS fused silica capillary column (30 m × 0.25 mm × 0.25 *μ*m) (Agilent Technologies, Santa Clara, CA, USA). Helium (purity 99.999%) served as carrier gas at a flow rate of 1 mL min^−1^. To ensure rapid and good separation of all the target compounds, a programmed temperature was employed. The initial column temperature was held at 60°C, and then to 150°C at 30°C min^−1^, and finally increased to 290°C at 10°C min^−1^, with hold time of 6 min. The injection port temperature was set at 280°C and the injection mode was in splitless mode.

In this study, the mass spectrometer was performed with an EI source in selected ion monitoring (SIM) mode. The electron energy was 70 eV with the temperature of the ion source and transfer line at 230°C and 280°C, respectively. The solvent delay was setting as 7 min. In the NIST search library, higher abundance of sub-ions was selected as qualitative ions and quantitative ions (Table 1).

## 3. Results and Discussion

### 3.1. Surface Structure of the Fiber

**Figure 1:**
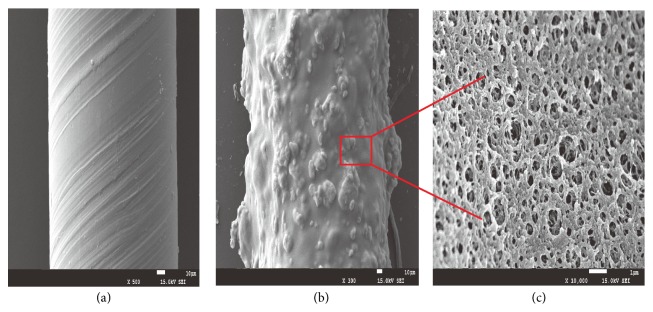
SEM images of the MWCNTs coated fiber, (a) the image of the stainless steel wire, (b) the image of the coated fiber's surface, and (c) the image of the coated fiber's inner structure.

Carbon nanotubes have the advantages of large specific surface area, good chemical stability, superior mechanical strength, and high heat resistance and can set off *π*-*π* interactions [21, 22]. Therefore, they have a promising application potential in the extraction field, for example, as SPME fiber. In order to investigate the surface properties of MWCNTs coated SPME fibers, seven pyrethroid pesticides were extracted with the proposed fibers and the uncoated stainless steel wires under the same experimental conditions. The results showed that the MWCNTs coated SPME fibers have a strong adsorption ability. Additionally, scanning electron microscopy (SEM) was employed to further explore the surface characteristics of the proposed fiber, as shown in Figure 1. It could be seen from Figure 1(b) that the multiwalled carbon nanotubes were uniformly attached on the surface of the stainless steel wire and were semiexposed. Figure 1(c) showed that the typical porous structure of MWCNTs coating was formed on the fiber surface, which resulted in high surface area of the coating, thereby offering extraordinary adsorption capacity. And the thickness of the coating is about 70 *μ*m.

### 3.2. Optimization of GC-MS Conditions

**Figure 2:**
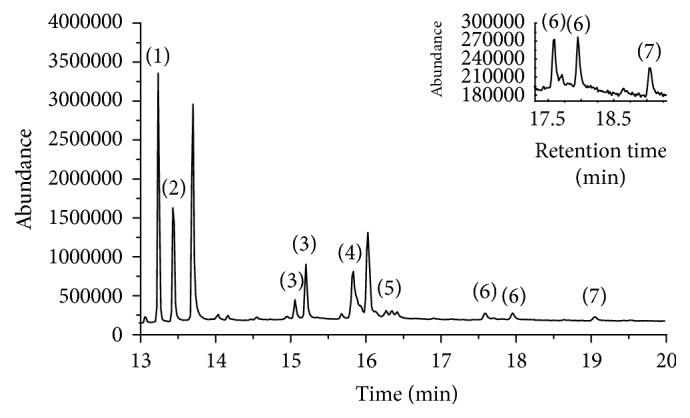
GC-MS chromatogram of the mixed standard solution of 7 pyrethroids. (1) bifenthrin, (2) fenpropathrin, (3) permethrin, (4) cyfluthrin, (5) cypermethrin, (6) fenvalerate, and (7) deltamethrin.

Tea has a challenging matrix, containing pigments, caffeine, and other impurities, which would interfere with the detection of the target compounds. In order to improve the accuracy and sensitivity of the method, it is necessary to select the appropriate mass spectrometry conditions. Under optimized GC-MS conditions, the chromatogram of the seven pyrethroids was shown in Figure 2.

### 3.3. Optimization of SPME Conditions

There are several main factors affecting SPME extraction and desorption efficiency, including stir rate, desorption temperature, and time, as well as extraction time. In this study, these factors were optimized as follows: the blank sample was spiked with the stock solution to obtain a final concentration of 20 ng mL^−1^. The analytes were extracted under different SPME conditions and analyzed by GC-MS. The optimal conditions were selected according to the peak area of the analytes.

#### 3.3.1. Optimization of Extraction Time

**Figure 3:**
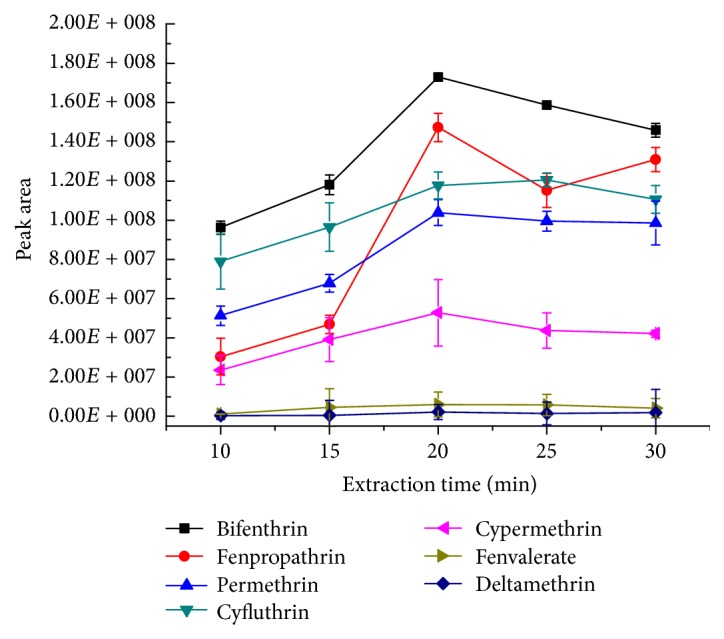
The effect of the extraction time on the extraction efficiency (*n* = 3). The standard mixture concentration: 20 ng mL^−1^, stirring rate: 750 rpm, desorption temperature: 280°C, and desorption time: 5 min.

According to the digital model of direct SPME and the dynamic adsorption model of nonbalance solid phase microextraction, the extraction efficiency is proportional to concentration of the target compound when other conditions are constant [23, 24]. In this study, analytes were extracted for 10, 15, 20, 25, and 30 min, respectively, with a stirring rate of 750 rpm and desorbing for 5 min at 280°C. The peak area of the analytes was used as the reference index. Each experiment was repeated for three times. As shown in Figure 3, the amount of the target pyrethroids increased with the increase of the extraction time. When the extraction time was 20 min, the extraction amount of the analytes reached the maximum and tended to be balanced. Therefore, taking into account the extraction efficiency and the analytical time, the extraction time of 20 min was selected.

#### 3.3.2. Optimization of Stirring Rate

**Figure 4:**
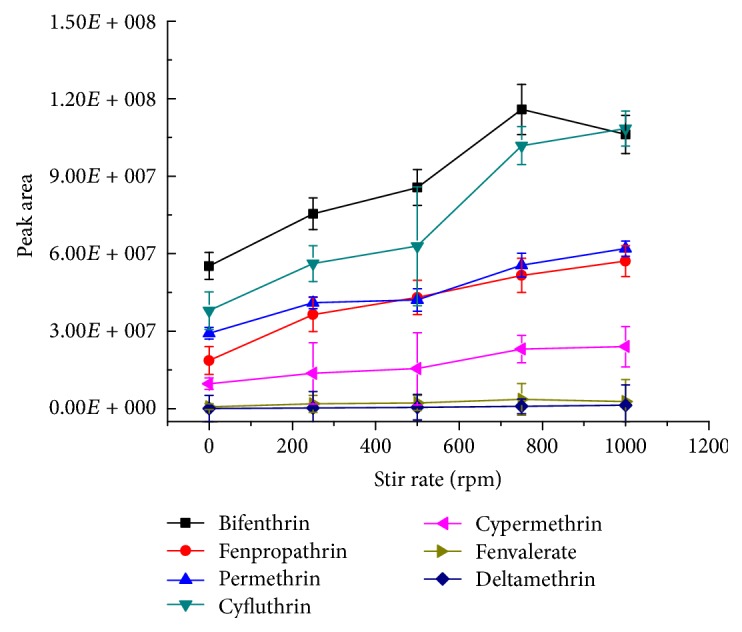
The effect of the stirring rate on the extraction efficiency (*n* = 3). The standard mixture concentration: 20 ng mL^−1^, extraction time: 20 min, desorption temperature: 280°C, and desorption time: 5 min.

The essence of SPME is to extract the analytes from the sample solution to the stationary phase. During the extraction process, the analytes in the solution diffused from the solid-liquid layer to the surface of the solid phase coating. Thus, the appropriate agitation facilitates the diffusion of molecules in the solution to improve the extraction efficiency [25]. The extraction efficiency of 7 pyrethroids was studied with stirring rates ranging from 0 to 1250 rpm when the other conditions were fixed, including extraction time of 20 min and desorption at 280°C for 5 min. Each experiment was repeated for three times. As shown in Figure 4, the peak area of the analytes increased as the stirring rate increased. Until 750 rpm, the peak area of the analytes reached the maximum. If the stirring rate continued to rise, it was prone to depression in the center of the liquid surface, which not only affected the normal operation of the experiments, but also led to a decrease of the extraction efficiency. Thus, 750 rpm was chosen as the stirring rate for subsequent experiments.

#### 3.3.3. Optimization of Desorption Temperature

**Figure 5:**
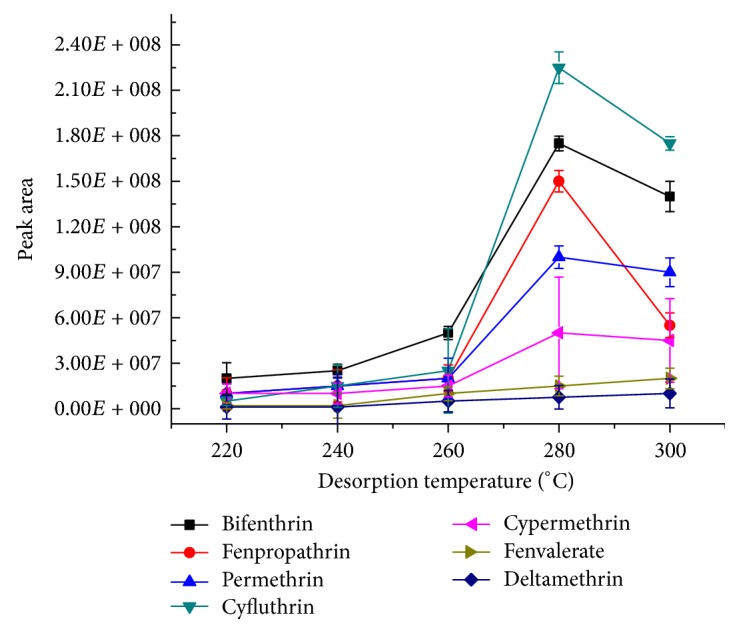
The effect of the temperature on the desorption efficiency (*n* = 3). The standard mixture concentration: 20 ng mL^−1^, stirring rate: 750 rpm, extraction time: 20 min, and desorption time: 5 min.

In SPME-GC-MS detection, if the desorption temperature is too low, the analytes cannot be effectively desorbed, and the carry-over would exhibit an adverse effect on both the results and the follow-up experiments, while if the temperature is too high, it might make the analytes decomposed and at the same time also affect the service life of SPME fibers [15, 26]. The influence of desorption temperature on the desorption efficiency for 7 pyrethroids was investigated in the range of 220–300°C. The other conditions were as follows: the stir rate was 750 rpm and the extraction time was 20 min with desorption for 5 min. Each experiment was repeated for three times. As Figure 5 shows, the elevated temperature facilitated the effective desorption of the analytes. The peak area of each analyte increased with the increase of the desorption temperature when it was in the range of 220–280°C. When the desorption temperature was increased to 300°C, the peak area of some of the analytes did not increase significantly and that of others decreased considerably, indicating that the analytes had been completely desorbed at 280°C. Therefore, 280°C was selected as the desorption temperature in subsequent experiments.

#### 3.3.4. Optimization of Desorption Time

**Figure 6:**
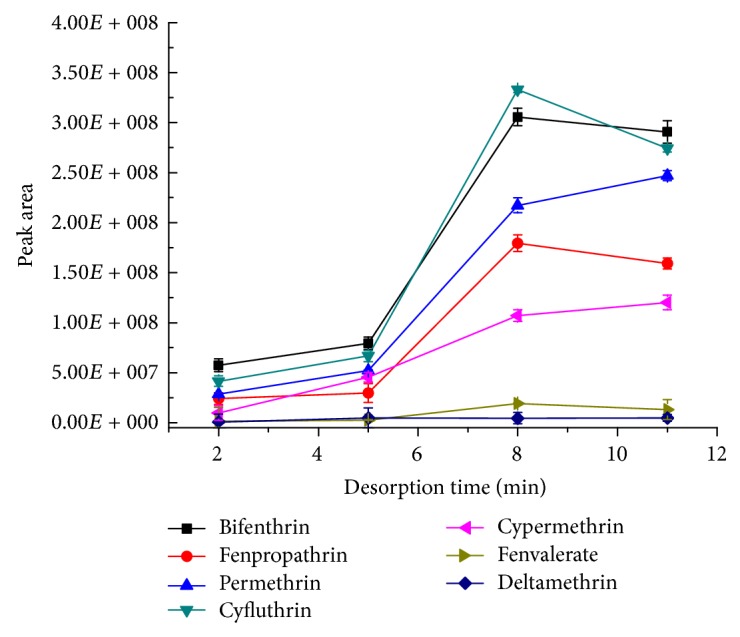
The effect of the desorption time on the desorption efficiency (*n* = 3). The standard mixture concentration: 20 ng mL^−1^, stirring rate: 750 rpm, extraction time: 20 min, and desorption temperature: 280°C.

In order to investigate the effect of desorption time, the analytes were determined when the desorption time was 2, 5, 8, and 11 min, respectively, under the conditions of extraction for 20 min with a stir rate of 750 rpm and desorption at 280°C. Each experiment was repeated for three times. As shown in Figure 6, when the desorption time was 2–8 min, the peak area of the analytes increased with the desorption time. However, when the desorption time continued to increase to 11 min, the peak area of most analytes did not change significantly. Therefore, 8 min was selected as the desorption time.

### 3.4. Method Validation

#### 3.4.1. Regression Equation, Linear Range, and Detection Limits

A standard series of 1, 5, 10, 30, and 50 ng mL^−1^ was prepared in the blank tea sample, and the assay was carried out in accordance with the procedure of 2.4. The standard curves were plotted with the peak area as the ordinate (*y*) and the mass concentration of the standard solution as the abscissa (*x*, ng mL^−1^). The limits of detection (LODs) and the limits of quantification (LOQs) are calculated on the basis of the 3-fold and 10-fold baseline noise, respectively. The results were presented in Table 2. There was a good linear relationship for bifenthrin, fenpropathrin, permethrin, and cyfluthrin in the range of 1 to 50 ng mL^−1^ and for cypermethrin, fenvalerate, and deltamethrin in the range of 5 to 50 ng mL^−1^, with the correlation coefficients (*r*) in 0.9948–0.9999. The detection limits were 0.12–1.65 ng mL^−1^, and the limits of quantification were 0.36–4.95 ng mL^−1^.

#### 3.4.2. Recovery and Precision of the Method

Under the optimized pretreatment and chromatographic conditions, the blank sample was spiked with the stock standard mixture to obtain a final concentration of 5, 10, and 50 ng mL^−1^, respectively, and then treated with MWCNTs coated SPME and analyzed by GC-MS. The recoveries were calculated according to the ratio of the determined concentration and the actual value. Each experiment was determined for 6 times. The results were shown in Table 3. The average recoveries ranged from 79.3% to 126% and the RSDs were less than 15%, indicating the favorable potential of the proposed method to be implemented in real application.

#### 3.4.3. Determination of Commercial Samples

**Figure 7:**
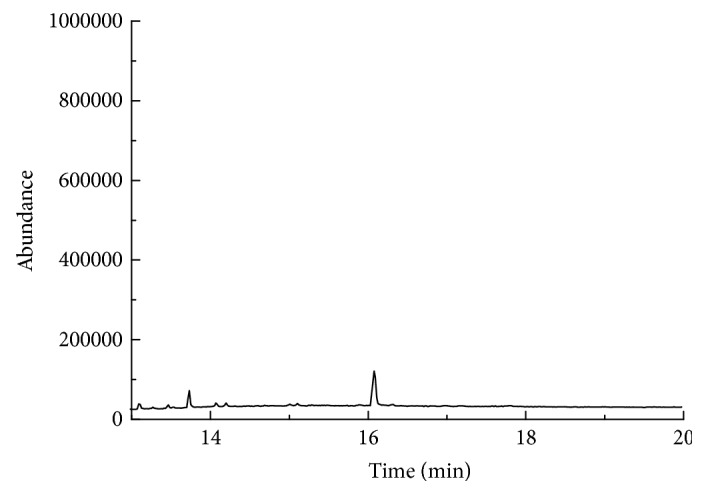
The chromatograms of the Maofeng Tea.

**Figure 8:**
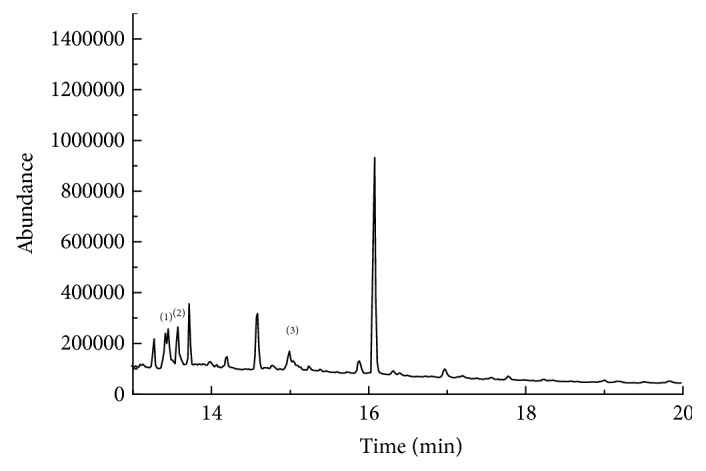
The chromatograms of the jasmine tea, (1) bifenthrin, (2) fenpropathrin, and (3) permethrin.

**Figure 9:**
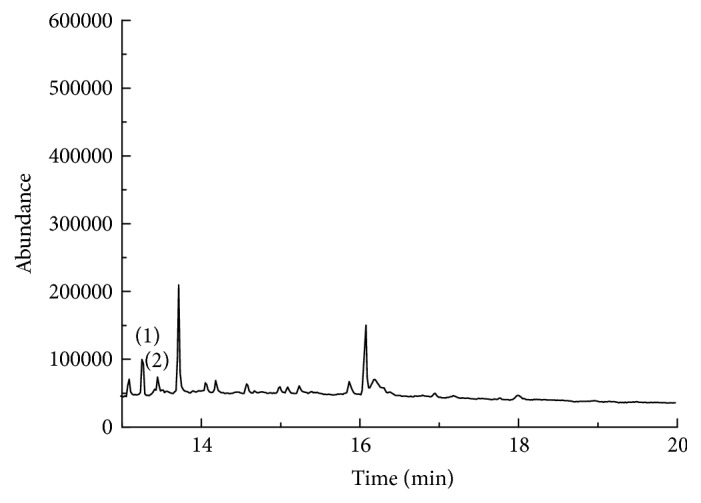
The chromatograms of the green tea, (1) bifenthrin and (2) fenpropathrin.

The proposed method was employed for the determination of 7 pyrethroid residues in tea brew samples made from different brands of tea randomly purchased from the local markets, and the results were shown in Figures 7–9. Bifenthrin, fenpropathrin, and permethrin were detected, although none reached the limit of quantification (the former two were found in jasmine tea and green tea, respectively, and the third one was only found in jasmine tea). Kang [27] determined 16 pesticide residues including fenpropathrin, cypermethrin, deltamethrin, fenvalerate, and lambda-cyhalothrin in tea brew by gas chromatography-electron capture detector (GC-ECD) after extracted by ethyl acetate for 3 times combining with LC-Florisil SPE column cleanup. The linear ranges and the recoveries of the method for the pyrethroids were in the range of 10 ng mL^−1^–1000 ng mL^−1^ and 63.1%–105.2%, respectively. When compared with this method, our method is more sensitive, is easier to operate, and consumes less organic reagents.

## 4. Conclusions

A multiwalled carbon nanotube coated SPME fiber was prepared by using stainless steel wire as the coating carrier and PAN as the adhesive. The SPME sampling combined with GC-MS analysis was employed to determine seven pyrethroids in tea brew samples. The proposed MWCNTs SPME not only has the advantages of fastness, efficiency, and convenience, but also can overcome the defects of the commercial fiber, such as ease of being broken and short service life as well as expensive price. The method is simple, rapid, and sensitive and with low consumption of organic reagents, which is suitable for the detection of pyrethroid pesticides in tea brew.

## Figures and Tables

**Table 1:** The retention time, monitoring ions, and quantitative ions of 7 pyrethroids.

Peak number	Pesticides	Retention time (min)	Quantitative ions (*m/z*)	Monitoring ions (*m/z*)
(1)	Bifenthrin	13.25	181.0	166.0, 182.0, 165.0
(2)	Fenpropathrin	13.45	349.0	141.0, 265.0, 209.0
(3)	Permethrin	15.10	182.0	163.0, 91.0, 165.0
(4)	Cyfluthrin	15.89	163.0	206.1, 226.1, 165.0
(5)	Cypermethrin	16.40	181.0	163.0, 209.0, 91.0
(6)	Fenvalerate	17.6, 18.02	125.0	167.1, 181.0, 225.0
(7)	Deltamethrin	19.07	253.0	251.0, 255.0, 181.0

**Table 2:** The linear ranges, correlation coefficients, LODs, and LOQs of the method.

Pesticides	Linear range/(ng mL^−1^)	Regression equation	Correlation coefficient	LOD/(ng mL^−1^)	LOQ/(ng mL^−1^)
Bifenthrin	1–50	*Y* = (2 × 10^6^ ± 5 × 10^4^)*x* + (4 × 10^6^ ± 2 × 10^5^)	0.9978	0.12	0.36
Fenpropathrin	1–50	*Y* = (9 × 10^5^ ± 5 × 10^4^)*x* + (2 × 10^6^ ± 2 × 10^5^)	0.9997	0.30	0.90
Permethrin	1–50	*Y* = (8 × 10^5^ ± 6 × 10^4^)*x* + (1 × 10^6^ ± 1 × 10^5^)	0.9999	0.33	0.99
Cyfluthrin	1–50	*Y* = (4 × 10^6^ ± 2 × 10^5^)*x* − (4 × 10^6^ ± 3 × 10^5^)	0.9999	0.24	0.72
Cypermethrin	5–50	*Y* = (4 × 10^5^ ± 4 × 10^4^)*x* + (9 × 10^5^ ± 1 × 10^5^)	0.9991	0.60	1.80
Fenvalerate	5–50	*Y* = (6 × 10^4^ ± 4 × 10^3^)*x* + (1 × 10^5^ ± 1 × 10^4^)	0.9997	0.99	2.97
Deltamethrin	5–50	*Y* = (9 × 10^4^ ± 6 × 10^3^)*x* + (1 × 10^5^ ± 1 × 10^4^)	0.9948	1.65	4.95

**Table 3:** The recovery and RSDs of the method.

Pesticides	Background/(ng mL^−1^)	Add/(ng mL^−1^)	Found/(ng mL^−1^)	Recovery/(%)	RSD/(%)
Bifenthrin	ND^a^	5.00	6.29	126	9.9
		10.00	10.02	100	2.5
		50.00	47.8	95.6	2.1
Fenpropathrin	ND	5.00	5.60	112	12.1
		10.00	7.93	79.3	13.9
		50.00	45.6	91.2	6.2
Permethrin	ND	5.00	5.89	118	12.6
		10.00	10.27	103	4.8
		50.00	50.28	101	2.0
Cyfluthrin	ND	5.00	4.73	94.6	14.4
		10.00	9.75	97.6	4.1
		50.00	51.96	104	2.9
Cypermethrin	ND	5.00	5.61	112	7.1
		10.00	10.83	108	3.5
		50.00	49.80	99.6	5.7
Fenvalerate	ND	5.00	4.60	92.1	8.5
		10.00	10.25	102	11.5
		50.00	48.41	96.8	1.3
Deltamethrin	ND	5.00	5.53	110	10.6
		10.00	9.76	97.6	7.2
		50.00	50.96	102	6.6

^a^Not detected.
